# Hypoxia Inhibits Hypertrophic Differentiation and Endochondral Ossification in Explanted Tibiae

**DOI:** 10.1371/journal.pone.0049896

**Published:** 2012-11-21

**Authors:** Jeroen C. H. Leijten, Liliana S. Moreira Teixeira, Ellie B. M. Landman, Clemens A. van Blitterswijk, Marcel Karperien

**Affiliations:** 1 Department of Developmental BioEngineering, MIRA Institute for Biomedical Technology and Technical Medicine, Faculty of Science and Technology, University of Twente, Enschede, The Netherlands; 2 Department of Tissue Engineering, MIRA Institute for Biomedical Technology and Technical Medicine, Faculty of Science and Technology, University of Twente, Enschede, The Netherlands; University of Western Ontario, Canada

## Abstract

**Purpose:**

Hypertrophic differentiation of growth plate chondrocytes induces angiogenesis which alleviates hypoxia normally present in cartilage. In the current study, we aim to determine whether alleviation of hypoxia is merely a downstream effect of hypertrophic differentiation as previously described or whether alleviation of hypoxia and consequent changes in oxygen tension mediated signaling events also plays an active role in regulating the hypertrophic differentiation process itself.

**Materials and Methods:**

Fetal mouse tibiae (E17.5) explants were cultured up to 21 days under normoxic or hypoxic conditions (21% and 2.5% oxygen respectively). Tibiae were analyzed on growth kinetics, histology, gene expression and protein secretion.

**Results:**

The oxygen level had a strong influence on the development of explanted fetal tibiae. Compared to hypoxia, normoxia increased the length of the tibiae, length of the hypertrophic zone, calcification of the cartilage and mRNA levels of hypertrophic differentiation-related genes e.g. *MMP9*, *MMP13*, *RUNX2*, *COL10A1* and *ALPL*. Compared to normoxia, hypoxia increased the size of the cartilaginous epiphysis, length of the resting zone, calcification of the bone and mRNA levels of hyaline cartilage-related genes e.g. *ACAN*, *COL2A1* and *SOX9*. Additionally, hypoxia enhanced the mRNA and protein expression of the secreted articular cartilage markers GREM1, FRZB and DKK1, which are able to inhibit hypertrophic differentiation.

**Conclusions:**

Collectively our data suggests that oxygen levels play an active role in the regulation of hypertrophic differentiation of hyaline chondrocytes. Normoxia stimulates hypertrophic differentiation evidenced by the expression of hypertrophic differentiation related genes. In contrast, hypoxia suppresses hypertrophic differentiation of chondrocytes, which might be at least partially explained by the induction of GREM1, FRZB and DKK1 expression.

## Introduction

Longitudinal growth of long bones is a tightly regulated process that is driven by hypertrophic differentiation and endochondral ossification of hyaline cartilage [1]. During maturation, the cartilaginous ends of long bones can be divided into three general zones: the resting, proliferative and hypertrophic zone. The resting zone is located closest to the ends of the diarthrodial long bones and is populated by small and round chondrocytes. Adjacent to the resting zone is the proliferative zone, which is characterized by vertical columns of actively proliferating chondrocytes. At the end of the proliferative zone, chondrocytes start maturing into terminally differentiated enlarged chondrocytes, which are located in the hypertrophic zone. Before hypertrophic chondrocytes undergo apoptosis they partially degrade and mineralize the extracellular matrix. Additionally, hypertrophic chondrocytes produce large amounts of angiogenic factors, such as vascular endothelial growth factor (Vegf) that recruits invading blood vessels into the hypertrophic cartilage [2]. This not only allows for the infiltration of amongst others bone forming cells, but also the alleviation of hypoxic stress (less than 5% oxygen) that occurs in most of the hyaline cartilage [3], [4].

**Figure 1:**
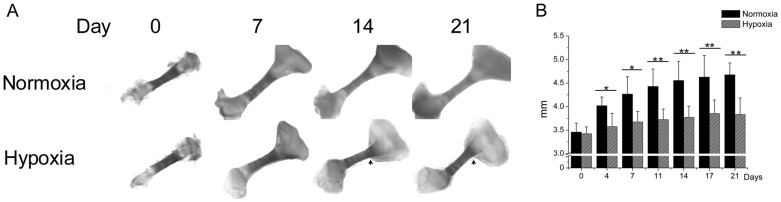
Explanted tibiae cultured 21 days under hypoxic or normoxic conditions. (A) Microphotographs of representative tibiae at different points in time. (B) Using image analysis the average tibiae lengths were calculated. (N = 18). * = P<0.05. ** = P<0.01.

Cells are able to adapt to hypoxia by means of the stabilization of hypoxia inducible transcription factors (Hifs) which subsequently influence the expression of genes that contain hypoxia responsive enhancer elements in their promoter region [5], [6]. Hypoxia regulated genes are amongst others involved in metabolism, bioenergetics and growth allowing cells to adapt to and survive in low oxygen tensions [7], [8], [9]. Additionally, hypoxia stimulates chondrogenic behavior in both mesenchymal stromal cells (MSCs) and chondrocytes [10], [11]. This stimulation occurs through both Sox9 dependent and independent pathways [12]. Alleviating hypoxia, in cultures of chondrogenically differentiated MSCs, results in a strong catabolic response [13]. Based on these lines of evidence, we hypothesized that oxygen tension is an active regulator of hypertrophic differentiation and consequently longitudinal bone growth.

In this study, we have investigated the effects of normoxia and hypoxia on longitudinal growth of mouse fetal long bones. We show that hypoxia, compared to normoxia, mitigates longitudinal bone growth by inhibiting hypertrophic differentiation and subsequent endochondral ossification. Furthermore, normoxia stimulates the calcification of the hypertrophic zone. Together our data suggest that oxygen tension, in particular the transition from hypoxia to normoxia, is an active and potent regulatory factor in endochondral ossification and longitudinal growth.

**Figure 2:**
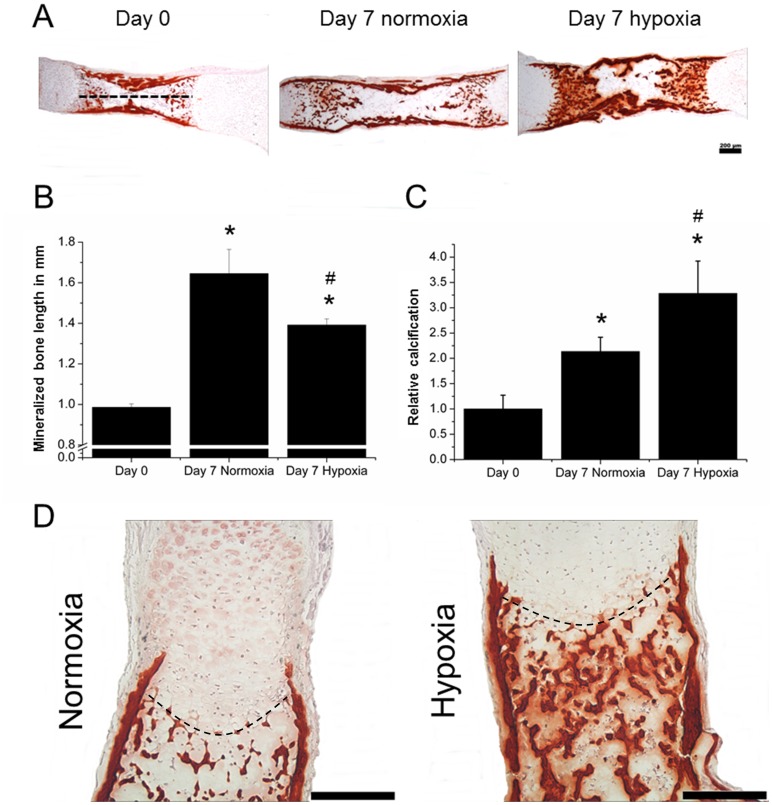
Histological analysis of calcification in explanted tibiae. (A) Midsagittal sections of tibiae were stained with Alizarin red after explantation or cultured seven days under hypoxic or normoxic conditions. (B) Image analysis was used to determine the length of the intensely calcified tissue, which was taken as the broken line indicated in ‘A’. (C) The area of calcification was used to determine relative calcification of the samples. (D) Higher magnification microphotographs were used to investigate the calcification of the hypertrophic cartilage that resides on top of the intensely stained bone. The dashed line represents the osteochondral interface. (N = 15). * = P<0.05 compared to freshly isolated tibiae. # = P<0.05 compared to normoxic condition of the same time point.

## Methods

### Ethics Statement

This study was performed by strictly following the recommendations of the guidelines of the general Dutch animal laboratory (GDL). The protocol was permitted by the Committee on the Ethics of Animal Experiments of the University of Utrecht (Permit Number: DEC 2009.III.09.093). All efforts were made to minimize suffering.

### Tibiae Organ Cultures

Tibiae were harvested from E17.5 fetal FVB-Type mice (Harlan) and cultured in medium consisting of α-MEM supplemented with 10% heat inactivated fetal bovine serum (Biowhittaker) and 100 U/ml penicillin and 100 mg/ml streptomycin (Gibco). Tibiae were either cultured in a low oxygen incubator at 2.5 percent oxygen (proox model C21, Biospherix) or at 21 percent oxygen (Sanyo) up to 21 days receiving twice a week fresh medium. Microphotographs of the growing tibiae were taken at multiple time points to determine their respective longitudinal growth (N = 18).

**Figure 3:**
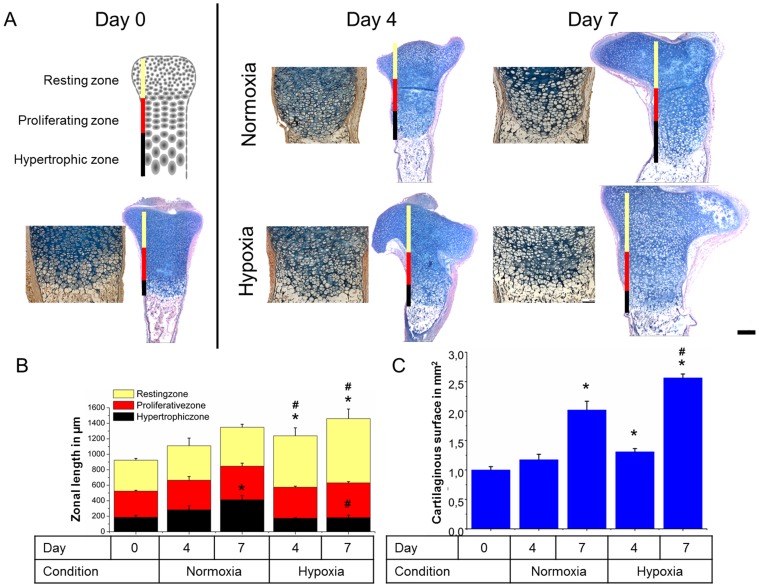
Histological analysis of zonal lengths in explanted tibiae. Midsagittal sections of tibiae were stained with Alcian blue and Nuclear fast red directly after explantation or after culture up to seven days under either hypoxic or normoxic conditions (A). Image analysis was used to measure the sizes of the different cartilaginous zones (B) and the surface of the cartilaginous area (C). (N = 15). * = P<0.05 compared to freshly isolated tibiae. # = P<0.05 compared to normoxic condition of the same time point.

### Histological Analysis

Tibiae were fixated using 10% buffered formalin, dehydrated using graded ethanols and embedded in paraffin. Specimen were longitudinally cut at 5 µm thickness using a microtome (Microm HM355S), deparaffinized in xylene and rehydrated by treatment with graded ethanols. Sections were stained with Alcian blue and Nuclear fast red (N = 15) or Alizarin red S (N = 15) according to standard procedures. For image analysis ImageJ software was used. Cartilage zones were judged as follows: small round chondrocytes were counted as the resting zone, stacked columnar chondrocytes were identified as the proliferative zone and the inflated chondrocytes following the proliferative zone were taken as hypertrophic zone. Length of the cartilaginous zones was determined as the shortest possible length as measured in midsaggital sections. The surface of the cartilaginous or bone area of midsaggital sections were quantified by calculating the blue or red surface area of the Alcian blue and Alizarin red S stained sections respectively (N = 15).

**Figure 4:**
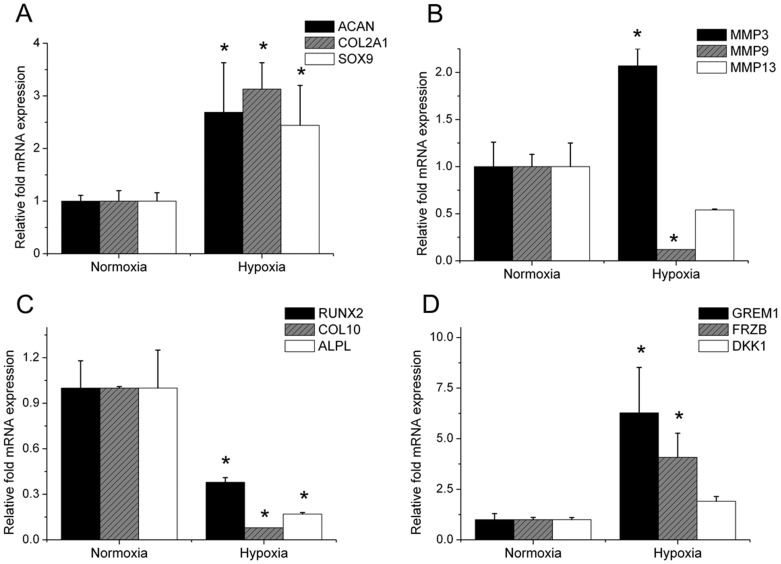
Gene expression in the cartilaginous heads of explanted tibiae. At 7 days mRNA was isolated and quantified using qPCR. Data are expressed as fold change relative to mRNA expression in normoxia Effect of hypoxia on mRNA expression of (A) typical cartilage markers, (B) cartilage degrading MMPs, (C) hypertrophic markers and (D) secreted Wnt and BMP antagonists able to inhibit hyptrophic differentiation. (N = 5). * = P<0.05 compared to hypoxia.

### Total RNA Isolation

Tibiae were washed in phosphate buffered saline. Both cartilaginous ends of the explanted tibiae were removed with a surgical blade under a stereomicroscope (Nikon SMZ800). Six cartilage specimens were pooled (N = 5) in a 2 ml tube and crushed using a Pellet stamp (Kontes) in the presence of Trizol (Invitrogen). Total RNA was isolated from the lysate using the Nucleospin® RNA II (Macherey-nagel) according to manufacturer’s protocol.

### Quantitative Real-time Reverse Transcriptase-polymerase Chain Reaction (qRT-PCR)

For each condition, one µg of total RNA was reverse transcribed into cDNA using the iScript^tm^ cDNA synthesis kit (BioRad) in accordance with the manufacturer’s instructions. Subsequently, expression of various genes was investigated by qRT-PCR. In short, 20 ng cDNA was amplified using sensimix (GC Biotech) on a MyIQ single color Real-time PCR detection system (BioRad) and analyzed with iQ^tm^5 optical system software (Biorad). The cDNA was denatured for 10 minutes followed by 45 cycles of 15 seconds 95°C, 20 seconds 60°C and 20 seconds of 72°C after which a melting curve was generated. Primer sequences are available upon request.

### Enzyme-linked Immunosorbent Assay (ELISA)

Medium with (N = 5) or without (N = 4) explanted tibiae was cultured up to 7 days in either hypoxic or normoxic conditions after which it was collected. Protein concentrations of secreted Frzb and Dkk1 were determined by ELISA following the manufacturer’s instructions (R&D Systems).

**Figure 5:**
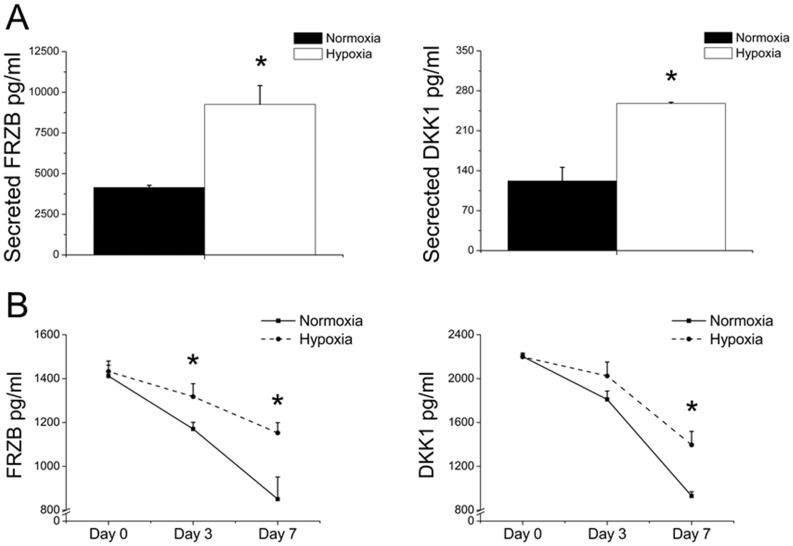
Effect of hypoxic and normoxic culture conditions on Frzb and Dkk1 protein levels. (A) Frzb and Dkk1 levels were quantified in the conditioned medium of tibiae, which were cultured for 7 days without receiving new medium in either hypoxia or normoxia (N = 5). (B) The effect of oxygen levels on Frzb and Dkk1 on protein activity over time was studied by exposing culture medium containing 10% fetal bovine serum to either hypoxia or normoxia for 7 days in 37°C. (N = 4). Frzb and Dkk1 protein levels were analyzed using ELISA. * = P<0.05 compared to normoxic condition of the same time point.

### Statistical Analysis

Statistical differences between two groups were analyzed using the Student’s *t*-test or one-way ANOVA. Statistical significance was set to a P<0.05 and indicated with an asterisk and/or hash-sign. Results are presented as mean of (how many repeats) ± standard deviation (SD).

## Results

### Normoxia Increases Longitudinal Growth

Biweekly macroscopical examination of explanted tibiae cultured up to 21 days in normoxia or hypoxia demonstrated longitudinal bone growth regardless in either condition (Figure 1A). Tibiae cultured under normoxic conditions grew significantly longer than tibiae cultured under hypoxic conditions (Figure 1B). The difference in longitudinal growth rate between both culture conditions was particularly dominant during the first week of the culture period. Remarkably, in hypoxia a marked increase in tissue growth was observed at the lateral sites of the osseous – cartilaginous interface (Figure 1, arrows). This suggested that the out-in gradient of oxygen was able to influence the shape of developing cartilage.

### Normoxia Stimulates Endochondral Ossification

To investigate whether the oxygen level dependent difference in growth was a result of endochondral ossification, midsagittal sections of tibiae cultured for 7 days under normoxic or hypoxic conditions were analysed histologically using Alizarin red S staining (Figure 2A). All tibiae increased in mineralized bone length, defined as the length between the cartilaginous ends. However, the area of mineralized bone of tibiae cultured under normoxic conditions was significantly longer than of tibiae cultured under hypoxic conditions (Figure 2B). This suggested that normoxia increased the pace of endochondral ossification. In contrast, semi-quantitative image analysis on midsagittal sections suggested that hypoxia resulted in higher absolute levels of calcification (Figure 2C). Interestingly, while the bone was more calcified under hypoxic conditions, evidenced amongst others by the increase in width of the bone collar, calcification of the hypertrophic cartilage was more intense in growth plates cultured under normoxic conditions (Figure 2D).

### Normoxia Increases Hypertrophic Zone’s Length

The length of the resting, proliferative and hypertrophic zone was determined based on Alcian blue and nuclear fast red stained midsaggital sections of tibiae cultured up to 7 days (Figure 3A). Freshly explanted uncultured tibiae showed similar zonal lengths compared to previously published observations [14]. All cultured tibiae showed a comparable increase in total cartilage length regardless of the culture conditions. However, we noted a remarkable difference in zonal organization of the primary growth plates: Tibiae cultured under normoxic conditions showed a progressive increase in length of the hypertrophic zone. In contrast, tibiae cultured under hypoxic conditions showed a progressive increase in the length of the resting zone (Figure 3B). Additionally, the total cartilaginous surface area of midsagittal sections was significantly smaller in tibiae cultured under normoxic conditions compared to hypoxic conditions (Figure 3C).

### Normoxia Increases Hypertrophy Markers’ mRNA Expression

The cartilaginous heads of tibiae demonstrated lower levels of chondrogenic genes such as *Acan*, *Col2a1* and *Sox9* when cultured under normoxic conditions then under hypoxic conditions (Figure 4A). Matrix metalloproteinases (Mmps) mRNA levels responded diversely to different oxygen levels; normoxia down regulated *Mmp3* mRNA, it up regulated *Mmp9* and tended to increase *Mmp13* mRNA levels (Figure 4B). The mRNA levels of genes related to hypertrophic chondrocytes such as *Runx2*, *Col10a1*, and *Alpl* were all expressed at a significantly higher level under normoxic culture conditions (Figure 4C). This suggested that hypoxia might be an important physiological factor preventing hypertrophic differentiation. Indeed, the mRNA levels of *Grem1* and *Frzb*, which we previously reported to be potent inhibitors of hypertrophic differentiation [15], were significantly up regulated under hypoxic conditions compared to normoxic conditions (Figure 4D).

### Normoxia Reduces Frzb and Dkk1 Protein Levels

To investigate the effect of the oxygen level on Frzb and Dkk1 protein expression, tibiae were cultured for 7 days after which their protein levels were quantified. In line with mRNA expression, Frzb and Dkk1 protein levels were significantly higher under hypoxic conditions compared to normoxic conditions (Figure 5A). Moreover, we investigated the effect of the oxygen level on Frzb and Dkk1 degradation. Fresh culture medium containing 10% fetal bovine serum was incubated at 37°C for up to 7 days in the absence of tibiae. This demonstrated that Frzb and Dkk1 protein levels declined more rapidly under normoxic conditions compared to hypoxic conditions (Figure 5B).

## Discussion

Longitudinal growth of long bones is driven by chondrocyte proliferation, chondrocyte hypertrophy and subsequent endochondral ossification of hyaline cartilage. This cartilage is predominantly avascular and its nutrient supply is dependent on diffusion from the surrounding tissue, being either the perichondrium or the blood vessels in the primary spongiosum. Consequently, out-in gradients of oxygen are present in hyaline cartilage [16]. The blood vessel formation at the osteochondral interface alleviates the terminally differentiated hypertrophic cartilage from its hypoxic stress [4], [9]. This effectively creates an oxygen gradient along the hypertrophic differentiating cartilage.

In this study, we have shown that oxygen levels were able to influence hypertrophic differentiation and subsequent endochondral ossification in explanted long bones cultured *ex vivo*. In particular, we demonstrated that normoxic conditions stimulate longitudinal growth compared to hypoxic conditions. This was, at least partly, explained by the difference in terminal differentiation; hypoxia retains chondrocytes in the resting zone while normoxia stimulates them to progress towards the hypertrophic zone. Indeed, the length of the mineralized bone grew significantly larger under normoxic conditions compared to hypoxic conditions. Previous reports typically described angiogenesis and the subsequent alleviation of hypoxia as a causal effect of hypertrophic differentiation. Here we report that the alleviation of hypoxia also plays an active role in regulating the process of hypertrophic differentiation itself.

Indeed, we observed a significantly lower expression of *Acan*, *Col2a1* and *Sox9* when explanted tibiae were cultured under normoxic conditions. Moreover, the shape of the tibiae became progressively more atypical under hypoxic conditions compared to normoxic conditions. This suggests that the out-in oxygen gradient, generated by the vascularized tissues surrounding the hyaline cartilage, as found *in vivo*, might contribute to defining the shape of the (cartilaginous ends of) long bones and controlling the direction of long bone elongation.

Blood vessels penetrate from the osteochondral regions into the hypertrophic zone. This process is driven by Vegf, which is expressed by hypertrophic chondrocytes and to a lesser extent by proliferative zone chondrocytes in response to the hypoxic conditions in the cartilage anlage [17]. This leads to vascularization of the cartilage, which results in normoxic conditions of the previously hypoxic cartilage. Compared to explants cultured in hypoxia, normoxic culture resulted in an increased expression of genes related to hypertrophic differentiation such as *Runx2*, *Col10a1*, *Mmp1* and *Mmp13* in the cartilaginous heads of the long bones [1], [13]. Increased expression of genes related to the terminal differentiation of hyaline cartilage coincided with an increased width of the hypertrophic zone in explants cultured in normoxia compared to explants cultured in hypoxia. In addition, we demonstrated that the mRNA levels of *Grem1* and *Frzb* as well as protein secretion of Frzb and Dkk1 were significantly lower under normoxia compared to hypoxia. Previously, we have shown that these three secreted antagonists are potent inhibitors of hypertrophic differentiation and subsequent endochondral ossification in explanted tibiae [15]. Indeed, in this study we observed an inverse correlation between the expression of these antagonists and hypertrophic differentiation. Therefore it is tempting to suggest that the effect of oxygen levels on hypertrophic differentiation of chondrocytes is at least in part mediated via the expression of these antagonists.

Tibiae contain a multitude of non-chondrocyte cell types, including osteoblasts and perichondral cells. It is possible that oxygen level mediated crosstalk occurs between the different cell types. However, studies in which Hifs were specifically (in)activated did not influence the longitudinal growth [9], [18]. Therefore, the observed effect on longitudinal growth is unlikely to be solely induced by a secondary cell source.

Taken together, we have demonstrated that the oxygen level is able to act as a potent regulator of chondrocyte’s hypertrophic differentiation and endochondral ossification of developing long bones.
